# Exploring the barriers and facilitators to non-medical prescribing experienced by pharmacists and physiotherapists, using focus groups

**DOI:** 10.1186/s12913-022-07559-5

**Published:** 2022-02-18

**Authors:** Emma Graham-Clarke, Alison Rushton, John Marriott

**Affiliations:** 1 School of Pharmacy, Institute of Clinical Sciences, College of Medical and Dental Sciences, University of Birmingham, Birmingham, UK; 2 School of Physical Therapy, Western University, London, Canada

**Keywords:** Barriers, Facilitators, Pharmacist, Physiotherapist, Prescribing

## Abstract

**Background:**

Non-medical prescribing (NMP) was introduced into the United Kingdom to enhance patient care and improve access to medicines. Early research indicated that not all non-medical prescribers utilised their qualification. A systematic review described 15 factors influencing NMP implementation. Findings from a recent linked Delphi study with independent physiotherapist and pharmacist prescribers achieved consensus for 1 barrier and 28 facilitators. However, item ranking differed for pharmacist and physiotherapist groups, suggesting facilitators and barriers to NMP differ depending on profession. The aim of this study was to further explore the lived experiences of NMP by pharmacists and physiotherapists.

**Method:**

Study design and analytical approach were guided by Interpretative Phenomenology Analysis principles. Focus groups (November and December 2020) used the ‘Zoom®﻿’ virtual platform with pharmacist and physiotherapist prescribers. Each focus group followed a topic guide, developed a priori based on the Delphi study results, and was audio recorded digitally. Transcripts underwent thematic analysis and data were visualised using a concept map and sunburst graph, and a table of illustrative quotes produced. Research trustworthiness was enhanced through critical discussion of the topic guide and study findings by the research group and by author reflexivity. The study is reported in line with COREQ guidelines.

**Results:**

Participants comprised three physiotherapists and seven pharmacists. Five themes were identified. The most frequently mentioned theme was ‘Staff’, and the subtheme ‘Clinical team’, describing the working relationship between participants and team members. The other themes were ‘Self’, ‘Governance’, ‘Practical aspects’ and ‘Patient care’. Important inter-dependencies were described between themes and subthemes, for example between ‘Governance’ and ‘Quality and Safety’. Differences were highlighted between the professions, some relating to the way each profession practises (for example decision making), others to the way the prescribing role had been established (for example administration support).

**Conclusions:**

The key finding of collaborative working with the clinical team emphasises its impact on successful implementation of NMP. Themes may be inter-dependent, and inter-profession differences were identified. Specifically designed prescribing roles were beneficial for participants. For full NMP benefits to be realised all aspects of such roles must be fully scoped, before recruiting or training non-medical prescribers.

**Supplementary Information:**

The online version contains supplementary material available at 10.1186/s12913-022-07559-5.

## Background

Non-medical prescribing (NMP) was introduced into the United Kingdom (UK) to enhance patient care and improve access to medicines [1]. Initially this enabled district nurses and health visitors to prescribe from a limited formulary [2] but in 1999, following the second Crown Report, the concept of independent and supplementary prescribing for nurses and other healthcare professionals was introduced [3]. Since then, the number of professions with independent prescribing rights has gradually increased and now includes nursing, optometry, pharmacy, podiatry, physiotherapy, paramedics and therapeutic radiography [4]. Demand exists for other professions to gain independent prescribing rights, with the Health Foundation commenting that until physician associates are able to prescribe independently, they will be limited in their activities [5]. Since the introduction of NMP, the UK National Health Service (NHS) has experienced increased patient demand, workforce shortage pressures, and funding shortfalls, driving policy emphasis to provision of streamlined care for patients, with NMP playing a pivotal role [6–8]. For example, prescribing physiotherapists, the first point of contact for many patients with musculoskeletal problems, are able to provide the complete treatment course without referral to other healthcare professionals [7, 9, 10]. A further example is that of pharmacists involved in the care of long term conditions [11]. These plans will be hindered if qualified non-medical prescribers are deterred, for whatever reason, from utilising their skills. Earlier research indicated that approximately 25% of Allied Health Professionals, qualified as prescribers, may not use this skill in comparison to 10% of qualified prescribing nurses [12, 13]. Establishing factors that facilitate or prevent NMP and investigating if these are generic to different NMP professions, or are professional, situational or person specific will aid NMP development.

A previous systematic review described 15 factors or themes that had the potential to influence the implementation of prescribing by non-medical professions [14]. It was noted that the majority of the included studies focused on prescribing by nurses, with the remainder addressing prescribing by pharmacists. The four most common themes identified included the influence of medical staff, the prescriber’s area of competence, the impact on their time and impact on service. No papers were found that reviewed the experiences of any other NMP profession. It is unclear whether or not the factors that affect prescribing by nurses and pharmacists are also experienced by other non-medical prescribing professions, or if they experience different factors.

To investigate this further a three round Delphi study investigating facilitators and barriers to independent non-medical prescribing was conducted with qualified independent prescribers from an established prescribing profession (pharmacy) and a newer, and relatively unexamined, prescribing profession (physiotherapy) [15]. The two professions were chosen as they have similar numbers of registrants in the UK (approximately 56,000), may work as individuals or in teams, and may work in all healthcare sectors [16, 17]. They differ in the length of time that each profession has had prescribing rights, with pharmacy gaining independent prescribing rights six years earlier than physiotherapy [18, 19]. Consensus was gained for 1 barrier and 28 facilitators, however, item ranking orders differed for the pharmacist and physiotherapist groups. This suggested that the facilitators and barriers to NMP differ depending on profession. However, it was possible that the differences arose from chance and did not accurately reflect experiences.

This paper presents the results of focus groups to further investigate the findings of the Delphi study, to explore if the findings reflected the experiences of pharmacist and physiotherapist prescribers, or if additional factors affecting implementation of NMP were also present. This would indicate how generalisable the Delphi study findings are to the wider pharmacist and physiotherapist prescribing populations.

### Aim

To further explore the lived experiences of non-medical prescribing by pharmacists and physiotherapists.

## Method

### Research team and reflexivity

EGC, JM and AR developed the study protocol and topic guide and EGC conducted the focus groups. EGC is a doctoral student, researching influences affecting NMP utilisation and inter-professional differences. The research question was prompted by her activity as an independent pharmacist prescriber, and her role as NMP lead for an acute NHS Trust in the Midlands. Her researcher standpoint is balanced by the other two researchers, neither of whom is a prescriber, but who have extensive research experience and represent the pharmacy and physiotherapy professions.

EGC acted as the contact point for participants during recruitment. Participants were made aware of the background to the research via the participant information sheet, issued at the time of recruitment, and this information was reinforced at the start of each focus group.

### Study design

The study design and analytical approach were guided by the principles of Interpretative Phenomenology Analysis (IPA) [20]. IPA acknowledges that the lived experience of each participant reflects their world view, and that interpretation is affected by the researcher’s own experiences. This study sought to understand how non-medical prescribers perceived their practice was affected by outside influences, whether procedural or people. Each participant will have had different formative experiences, shaping their view of NMP, and IPA will aid in interpretation of this, whilst recognising the potential influence of the lead researcher.

Focus groups enable discussion between participants on selected specific topics. The discussion and interaction between the participants allow ideas and views to be developed and refined, and thus provide a deeper understanding of the issues being considered [21, 22]. There is also the potential for unanticipated ideas to be expressed, supporting further understanding of the research topic [22]. Research indicates that 80% of ideas are generated within the first two or three focus groups, and these comprise the most frequently mentioned themes [23, 24]. Furthermore, Hennink describes focussed research questions requiring fewer focus groups to generate ideas than research questions where the issues are unknown [25]. A pragmatic approach to the groups was adopted, balancing available resources and the level of information anticipated from the closely defined topic guide [25]. Two focus groups were planned, using the ‘Zoom®﻿’ virtual platform (Zoom.us), hosted by the University of Birmingham. Each group was led by a moderator (EGC) and the conversation was audio recorded digitally, using the virtual platform record feature, and handwritten fieldnotes were taken. Each focus group followed a similar format of introduction, main discussion and closing stage, and followed an a priori developed topic guide [21, 26–28]. The topic guide was drafted by EGC, using the previous Delphi results as a guide, and debated within the research group to ensure that the guide was clear, followed a logical progression and was appropriate for the aim of the study (Additional File 1). The topics chosen were those where there were apparent differences in the Delphi results between the professions when reviewing the ranked statements by profession. The discussion was summarised after each topic and at the end of each focus group, enabling participants to comment and correct any misinterpretation.

### Choice of setting

Focus groups are conventionally run face to face, using a location suitable for researchers and participants. However, to reduce transmission of Covid-19, people were advised to physically distance themselves, to meet outdoors rather than inside and to wear face masks [29], making physical meetings difficult to conduct. Virtual focus groups have been previously reported, with researchers using a variety of techniques such as message boards and video conferencing, with cost of equipment (e.g., webcams) and programmes listed as potential disadvantages [30, 31]. The restrictions imposed to limit the spread of Covid-19 accelerated the widespread adoption of virtual meeting platforms such Zoom® for both work and social uses. Indeed, many participants in this study described the benefits of online meetings, indicating that many of the earlier challenges with virtual platforms, such as equipment availability, had been overcome. Table 1 lists potential advantages and disadvantages of physical (under Covid-19 restrictions) and virtual meetings. The assessment was made that, with the ongoing pandemic associated restrictions, the virtual platform was the most appropriate technique to enable the focus groups to be conducted.

**Table 1:** Comparison of physical and virtual meetings for focus groups

	**Physical meeting, under Covid-19 restrictions**	**Virtual meeting**
Advantages	● No special equipment required e.g., cameras ● Conversation and discussion can flow easily ● No specialist knowledge (e.g., computer literacy) required	● No travel required; participants may be able to join who would otherwise be time restricted ● Virtual platform includes record function ● Face masks may not be required, dependent on participant’s location ● Participants can join from any suitable location
Disadvantages	● Large room required to enable social distancing ● Face masks need to be worn, hiding facial expressions ● Recording equipment required ● Travel, and travel time, required to attend meeting location	● Only one person can speak at once, potentially stilting conversation ● Depends on internet connectivity ● Requires computer or smartphone or similar, with audio and camera ● Participants required to have basic computer literacy

### Participants and recruitment

Participants for the focus groups included independent prescribing pharmacists or physiotherapists working in primary or secondary care in the West Midlands region. No easily accessible list for pharmacist and physiotherapist independent prescribers was available and therefore participants were recruited indirectly using groups such as the United Kingdom Clinical Pharmacy Association and West Midlands NMP leads. An email, including study details, participant information sheet, screening questionnaire and contact email address, was sent to these groups and recipients were requested to forward the email to potential participants.

The number of qualified independent pharmacist and physiotherapist prescribers in the West Midlands region is unknown, as this information is recorded by individual healthcare providers, and not centrally. Therefore, the intention was to recruit 10 prescribing pharmacists and 10 prescribing physiotherapists, allowing for non-attendees, but providing sufficient participants for a meaningful discussion [21, 25, 32]. The literature on focus groups recommends a group size of 6 to 8 participants, with recommendations to over recruit by approximately 20% in case of non-attendance [21, 25, 32]. Participants were required to have obtained their prescribing qualification since the beginning of 2013 (when physiotherapists gained independent prescribing rights [19]), and the final selection was guided by the sample matrix in Table 2.

**Table 2:** Target sample matrix for focus group participants

**Criteria**	**Pharmacist**	**Physiotherapist**	**Years of professional practice**	
Profession	10	10	≤5	0-5
Length of time qualified as a prescriber:			6-10	0-5
≥12 months	1-6	1-6	11-15	0-5
<12 months	1-6	1-6	16-20	0-5
Main practice area:			>21	0-5
Primary Care	1-6	1-6		
Secondary care	1-6	1-6		

Participants were asked to sign and return a consent form, including consent to record the focus group, prior to the focus group being conducted. Recruitment was closed in October 2020.

### Ethical considerations

Ethical approval for the study was obtained from the University of Birmingham’s Science, Technology, Engineering and Mathematics Ethical Review Committee (ERN_19-1900) and all data were held securely in accordance with university policy. Participation was voluntary and participants were free to withdraw at any time, however they were made aware that if they had already participated in the discussion, then it would not be possible to remove their contribution. All participants gave written consent, including for digital audio recording, prior to the focus group. All recordings were transcribed verbatim and anonymised to ensure that participants, locations, or other identifiable information were removed, and participants were assigned an identification code.

### Data analysis

Digital transcripts of each conversation were produced by the virtual platform, and these were checked for accuracy, corrected, and verified by EGC. This process required repeated listening to the recording, hence ensuring all information was captured accurately, and permitting immersion in the data. Following transcription, data were imported into NVivo® 12 (QSR International) for thematic analysis [21, 33, 34]. The transcript for Focus Group One was read and reread to identify emergent themes and patterns, and coded line by line, with new codes created as themes emerged. The process was repeated for Focus Group Two, with further themes added as they emerged. Coding was an iterative process, with repeat reviewing of the coded data to ensure consistency and initial thoughts on the findings recorded using the NVivo memo function. Finally, the themes were reviewed and consolidated where appropriate. A codebook was produced to support the coding process. Data was visualised using a concept map of the major and minor themes and interdependencies, and a sunburst graph which depicted the frequency that themes were mentioned. Quotations illustrating each theme were presented as a table (Table 4). The initial coding was done by EGC, and the themes and hierarchy were discussed critically by the research team.

The study is reported in accordance with the COREQ statement (Additional file 2) [35].

## Results

Eighteen participants initially expressed an interest in participating in the focus groups. The recruitment window was extended, and further invitation emails sent to encourage further interest in participation, but the response remined low. The decision was taken to conduct the focus groups with the existing pool of potential participants, rather than risk a high dropout rate as participants were called to care for Covid-19 patients. Even with this approach, five potential participants who had previously expressed an interest failed to respond to the focus groups emails. A further three participants were excluded: two were ineligible, and dates were unsuitable for one, leaving ten participants. Three participants participated in Focus Group One and seven participated in Focus Group Two. Brief demographic data are included in Table 3. Focus Group One was held on 23 November 2020 in the evening and Focus Group Two on 3 December 2020 during the day, each lasting just over one hour.

**Table 3:** Brief participant demographic data

Participant ID	Profession	Practice area	Years qualified in profession	Active prescriber	Focus Group
FG1-P1	Pharmacist	Ward, secondary care	16–20	Yes	One
FG1-P2	Pharmacist	Clinic, secondary care	16–20	Yes	One
FG1-P3	Pharmacist	Ward, secondary care	6–10	Yes	One
FG2-P1	Physiotherapist	Clinic, primary care	16–20	Yes	Two
FG2-P2	Pharmacist	Clinic, secondary care	6–10	Yes	Two
FG2-P3	Physiotherapist	Ward, secondary care	16–20	No, temporarily stopped	Two
FG2-P4	Pharmacist	Ward, secondary care	16–20	Yes	Two
FG2-P5	Physiotherapist	Clinic, primary care	> 21	Yes	Two
FG2-P6	Pharmacist	Clinic, secondary care	11–15	Yes	Two
FG2-P7	Pharmacist	Ward, secondary care	11–15	No, never prescribed	Two

Initial coding was reviewed by EGC by reading the results for each node coded and the matrix tool in NVivo utilised to check that coding was appropriate. A concept map of themes was derived by EGC following coding of the transcripts, and the map and derived themes were debated by EGC, AR and JM to ensure they reflected participants views. After further discussion, the hierarchy and concept map were re-drawn to reflect the lived experiences of the participants more accurately. For example the original hierarchy did not contain a ‘self’ theme and hence ‘personal competence’ was grouped under ‘governance’ instead. However, as this quote highlights, ‘personal competence’ is derived from the participant’s views and feelings, not externally driven:

*‘…as long as it's, it's, something that, you know, you feel comfortable within your competence, because I think that's where sometimes, some of my colleagues have got more experience in sexual health, whereas I haven't so it might be something that I'll say ‘I'm not comfortable. I would refer you to this service’…’ FG1-P2*

Obsolete or duplicate codes were also removed, for example the original codebook included an ‘advisory role’ code, but on review the ‘team role’ code was deemed to be more appropriate.

**Fig. 1:**
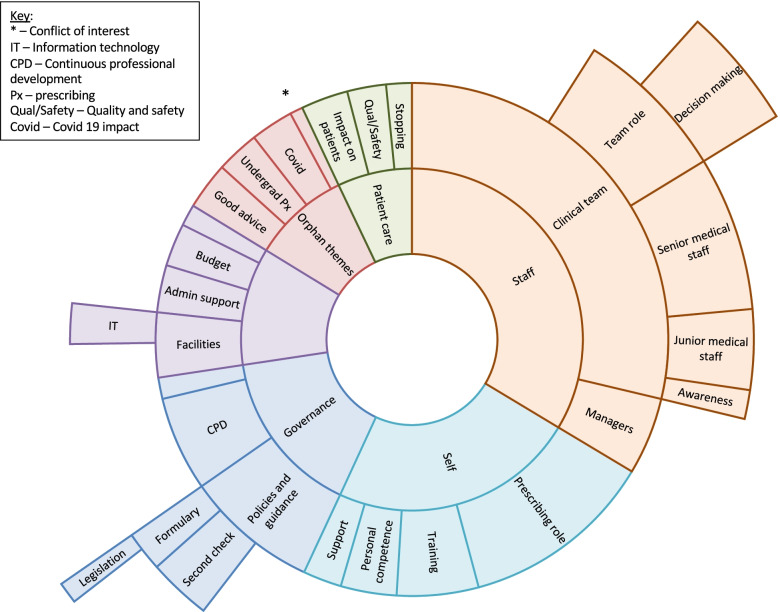
Sunburst chart depicting the themes and subthemes, and their relative importance as indicated by area of segment

Thematic analysis identified five themes each comprising several subthemes. Figure 1 depicts the themes as a sunburst chart. The size of each segment reflects the number of references to the item, and hence the relative importance of the topic to the participants. The inner ring contains the themes, with subthemes radiating out.

**Fig. 2:**
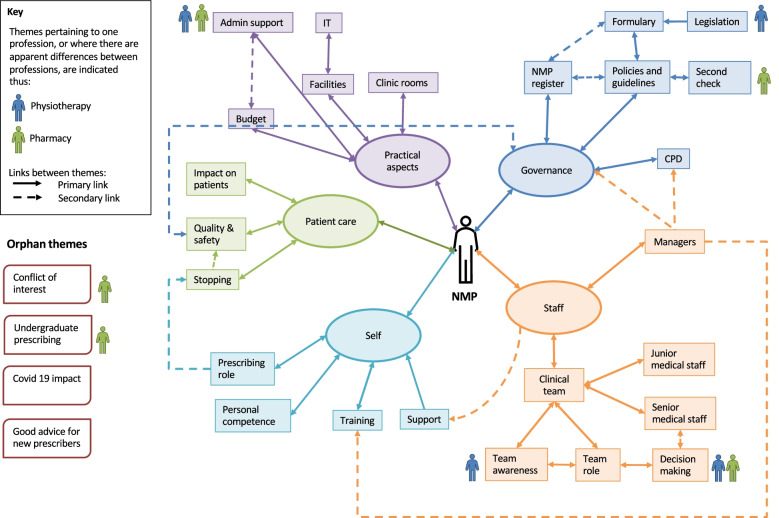
Concept map of hierarchical structure depicting interrelationship between themes and sub themes

Figure 2 is a concept map depicting the hierarchy and interrelationships between themes and subthemes. Table 4 lists the themes and sub themes, their code book descriptions, and illustrative quotes from the participants.

**Table 4:** Code book description of themes and sub themes, with illustrative quotes

Name	Description/Code book entry	Illustrative quotes
**Staff**	Overarching theme bringing all staff related themes together	
Clinical team	Prescribing within, or supported by, multidisciplinary team. Degree of integration into the team. Team or autonomous working	“I say that because I work in a really small MDT and I, and I work with consultants that because we're such a small team It's all first name terms, we can easily kind of have a dialogue and get hold of each other…” FG1-P2
		“I think, for us being able to prescribe has kind of made me more part of the medical team. Yeah, because they kind of see me as more similar to them. So, they've sort of accepted me a little bit more, if that makes sense?” FG2-P3
		“I sometimes feel like I'm not fully part of any one team because I sort of dip in and out of different teams.” FG2-P4
		“Um, for me, because we mostly work on our own and autonomously, a lot of the decisions fall with just myself with prescribing unless there is an issue, and then I would refer, would ask for advice from our specialist that we have meetings with every week.” FG2-P1
Junior medical staff	Working relationships with junior medical staff. Impact on junior medical staff workload. Potential for deskilling junior medical staff by prescribing. NMP teaching junior medical staff	“…so it is actually taking a lot of workload out of that system for the junior staff particularly so they can focus on things I can't do like bloods…” FG1-P3
		“I think the junior medics appreciate having a specific point of call. I, like, during the day I get often get bleeps and queries from all over the hospital about ‘oh I've got this patient on this and we want to switch them to this for discharge.” FG2-P2
		“…the junior doctors accept that it's normal to have an ACP, and they prescribe, and it's normal that they're from a variety of backgrounds, because that's what they've been exposed to.” FG2-P3
Senior medical staff	Working relationships with senior medical staff. Building trust between NMP and consultants. Constructive discussions with senior medical staff. Consultant concern regarding accountability	“The only thing I was gonna say is some of the consultants, particularly those when they’re sort of new to having an ACP around still feel that they are responsible and accountable for what you do.” FG2-P3
		“I think there is a shift now of um understanding as sort of more junior consultants have come through. Um, it's fine now, but I think this is, I mean, this has been happening over several years now. Now it's, it's much more accepted that physios don't just do exercises or patting on the back. (laughs)” FG2-P1
		“And I think the consultants probably work with me in a slightly different way. They tend to ask if they’ve got more complex queries or areas where there's less evidence for kind of my interpretation of it, and that tends to be more of a discussion” FG2-P2
Team awareness	Team awareness of prescribing role, or lack of awareness	“But as for junior doctors, I'm not even sure if they know that I'm a prescriber because I suppose I don’t go around saying ‘I'm a prescriber’.” FG2-P4
		“…my, my colleagues, that I've been working with for many years, have gone through that process with me because obviously they’ve been my supervisors and stuff, so, um yeah, it's kind of more the not, not the inexperienced but the ones that haven't been part of the team that are unaware of that.” FG2-P5
Team role	NMP role within the team. Includes team interest in prescribing or lack of, and the effect on the NMP workload and role. Also, is the team in a better position to prescribe than NMP or are NMP’s non-prescribing skills utilised more than prescribing	“I feel like I'm very much part of the team and they recognize my area of expertise and things like that (nods from FG2-P6). So, I think I feel like we work like well together. And it's, it's a really good job. I wouldn't want to give it to someone else!” FG2-P2
		“So, I work with consultants, with clinical specialist nurses as well and psychologists. … … So, you know, I don't think it's imperative for my role that I needed to, I need to prescribe. I need to have an understanding, though, of the, of the drugs because I work with, you know, other members that can prescribe. I suppose my skills are elsewhere.” FG2-P5
		“I sometimes feel amongst, that certainly some of the doctor teams, I work with, it's, it feels the opposite, and they very much want to palm off work. … … So, I haven't left on time for a long time … … a large part of that is because I'm constantly being stopped by nurses that now know I’m a prescriber and want me to write things up.” FG1-P3
Decision making	Who makes the initial prescribing decision—NMP or medical team? Does the NMP make full use of their taught skills or not? Outcome dependant on role within the team, and medical staff attitude	“So, I think they’re finding it useful to have like an extra prescriber who’s physically there, who can, if he says, ‘I want to increase this dose’, I can physically do the writing of that on the chart. Yes, I think, I mean in psychiatry it's usually the consultant who makes a decision about which medication to use, but they’re quite open to discussion about that and tweaking things.” FG2-P4
		“The thought I want to throw in is do you, do you sometimes feel though as if you are… … do you feel sometimes you just seen as the person just doing the writing of the prescription rather than doing the decision making of the prescription or, or is that … …is that accepted now so that we can make the full decision process?” FG1-P1
		“No, I'd say, I don't really find that find that I feel like I'm just kind of writing out somebody else instructions, either. so, any new antiretroviral it's always a very sort of team led decision.” FG1-P2
Managers	Impact on NMP. Support for NMP and NMP role and understanding of NMP role. Differences highlighted between prescribing and non-prescribing managers. Links with ‘CPD’, ‘Training’ and ‘Governance’ themes	“…my line manager is, had completed the prescribing course before me. So was very well versed in what it involved and what it could, how it could enhance my role and then has put me in a, in a position to use it in a really effective way.” FG1-P3
		“Yeah, I was gonna say so my line manager is nonclinical. They are from management background, so they have no real understanding of the role when they started. So, it was an explanation of the role and what it meant. Um, so they have quite limited understanding about the issues that might be involved.” FG2-P3
		“…because often like quite a few people have said your actual line manager doesn't have an understanding of what you might be doing clinically or the risks you might be taking.” FG2-P2
		“So, the new one, I think she's just happy that she's got a prescriber, because I was a first pharmacist prescriber in the team. … … she's always kind of offering me out to people, and ‘oh, FG2-P4 can come and do some clinics.’ But I think it's, I don't know if she understands the logistics and sort of how it'd be.” FG2-P4
**Self**	Overarching theme containing themes relating to the NMP themselves, their views and practice	
Personal competence	Personal competence around prescribing. When they refer on to someone else and which areas the NMP is comfortable to prescribe in	“Okay, yeah, I would just say I suppose know what your specialist area is. it's not that you can't ever prescribe off your, your limited formulary. But know what your limits are because I think I know people go, oh I’ve got to get a doctor to need to sign this, can you just sign this and you think well no, that’s not what I'm here to do.” FG2-P4
		“I just want to say I don't always necessarily agree with the prescribing of the consultants …. So, I tend to, I wouldn't prescribe that myself and I wouldn't rewrite that because then it's got my signature on it. … …I'll certainly prescribe what I'm happy with prescribing.” FG2-P4
		“…some of my colleagues have got more experience in sexual health, whereas I haven't so it might be something that I'll say ‘I'm not comfortable. I would refer you to this service’” FG1-P2
Prescribing Role	Role that prescribing plays within the job. Whether or not prescribing is an essential part of role. For existing jobs, who covers aspects of the existing role. Blurring, or clarity, between NMP and professional role aspects. Links with ‘Stopping’ theme	“…but I've never thought as a prescriber that I've ever been given that allocation of time to make sure that, you know, you can function in the role when you're doing clinics.” FG2-P6
		“I think it's probably easier, like my role was a new role and the expectation from the medical side was that this person would prescribe, whereas I think it's maybe slightly harder if you've got an existing role and then do it, because then you need to create the time to do it and some other part of your job has to go somewhere or to someone else, …” FG2-P2
		“So, you know, I don't think it's imperative for my role that I needed to, I need to prescribe.” FG2-P5
		“you know, we've taken on that role and, er, and, and, particularly sort of with more some more clinical competencies coming along we’re properly taking on newer roles’ um, but we need to make sure that, then the sort of traditional roles are either filled or taken up” FG1-P1
Support	Who, or when, to ask for help. Support from different areas and people. Use of networks for support. Links with ‘Staff’ theme	“…so I asked to have a professional kind of supervisor in a way, that I could go to if there were any issues and to make sure that I sort of safety netted myself…” FG2-P3
		“…so if I see a patient that's more complex or slightly unusual, that doesn't fit the usual pattern, then I can just catch him between patients or if it's less urgent I'll discuss that patient with him at the end of the clinic.” FG2-P2
Training	Ease of access to training course, or challenges. Personal development during the training course. Medical supervisor support and change in working relationship resulting from the course. Benefit of an area of expertise when undergoing the course	“And it comes with a whole host of other skills, isn't it, that you're learning as well. It's about, you know, your history taking, examination skills and a whole host of other skills as well” FG1-P2
		“…my line manager has supported me in terms of the prescribing, are quite happy for me to go on the course…” FG2-P7
		“The, the course itself really enhanced my practice because although it was an absolutely mission, getting the hours in around full time work … … I really do think it helped me hugely because the um so spending time one on one with consultants in their clinics, for example. … …I think they then saw me as more of an integrated member of the team.” FG1-P3
		So, so that, yeah, that took a little while to actually get the agreement for the, for the for the funding to, to do that, because they couldn't just quite understand what the purpose of it is, yeah.” FG1-P1
**Governance**	Overarching theme which incorporates aspects such as policies and guidance, formulary, legislation etc. Certain aspects have cross links with other themes, for example ‘CPD’ links with ‘Managers’	
CPD	Support to attend CPD, including provision of time and funding. Availability of CPD within work environment and outside. Self-directed or directed by manager/organisation. Benefits of web-based meetings and conferences	“If it's something that costs money, then it can be a bit more difficult to arrange” FG2-P2
		“I book my own diary. Um so I if I need to do CPD, I can just build it in but also, I can attend the monthly non-medical prescribing meetings that we have at our trust, which also has an element of CPD as part of the meetings…” FG2-P1
		“In fact, this, this Covid’s been a wonder, because I can actually get to all these webinars, instead of having to go to stuff.” FG1-P3
		“…we have a monthly pharmacists clinical supervision meeting and also the monthly NMP supervision meeting so there’s various things that built in that you can do CPD but obviously you can go off and do more reading and things in your own time as well.” FG2-P4
NMP register	Ease of registering as an NMP with organisation. Maintenance of registration. Amending register entry	“I think I've signed a form but I’m really not quite sure what actually happened with it.” FG1-P1
		“Um, yeah, I think it's just that attitude in Trusts that NMPs have to jump through many hoops, don't they, and they have to, once you’re qualified, you can't just start prescribing, you have to then go to a committee to be stamped, and they have to prove evidence of things.” FG2-P4
Policies and guidance	Policies and guidelines relating to NMP: the clarity and value of them, or absence of them. The support given by them	“We’ve got a non-medical prescribing policy, haven’t we, but it seems quite a vague in its limitations.” FG1-P3
		“So, I think our, our, I guess, policy and things are quite clear that you, you have like an allocated clinical supervisor, as such, and you should be meeting them like once or twice a year to kind of check in…” FG2-P2
Formulary	Personal or organisation formulary affecting NMP role	“And I had to give a list of a maximum of 10 drugs I could prescribe…” FG2-P3
		“Have your small, very small personal formulary and build up so that you're prescribing really well, just a small handful of drugs to begin with and then slowly expand it.” FG2-P1
Legislation	Effect of legislation on prescribing, particularly affecting physiotherapists	“…the main sort of medications you would have used, we weren't able to as physio’s, um so, your gabapentin, obviously, and pregabalin’s changed, codeine we weren’t, we weren’t able to, so co-codamol we weren’t able to prescribe.” FG2-P5
Second check	Value of second check by a pharmacist when prescribing. Mentioned by pharmacist prescribers within the context of safety	“I think, I think, I think it's probably both true, isn't it, because FG1-P2 you've got a very, very specialist role and, and so have, I and we can both, our prescriptions will go and be checked by somebody else…” FG1-P1
		“…in reality, a lot of TTOs don't come through pharmacy, a lot of them are nurse lead TTOs. so I am, I then become the technician, pharmacist and pharmacist prescriber for that patient. I’m the only pharmacist contact, pharmacy contact, that that patient sees or gets and I'm prescribing as part of that role, which probably opens me up to some risk of error in terms of not getting a second check.” FG1-P3
**Practical aspects**	Overarching theme which incorporates aspects such as access to clinic rooms, budget, admin support etc	
Admin support	Availability of administrative support. Allocated time to complete NMP associated administration	“…like some weeks I might do three clinic sessions, which then generates a significant amount of after clinic work but there's no, there’s not the recognition that you also need time to do that work.” FG2-P2
		“I do a mixture of home visits and clinics and we have as much admin time as we need really.” FG2-P1
Budget	For post and equipment. Source of budget – single or multiple departments	“Because I think, particularly I think in pharmacy, they look to get that financial support from the directorates that they're prescribing for rather than just coming out of the pharmacy budget.” FG2-P6
		“…I’ve had to sort of justify why I need a laptop, why I need a mobile phone, why I need headset and camera…… my role is jointly funded by pharmacy and the ID directorate as well so it's the barrier I find sometimes is, is well which budget is it going to come from.” FG1-P2
Clinic rooms	Access to, and availability of, clinic rooms	“In our trust, clinic rooms are at a premium. They are really struggling for space and that has been one of, one of the barriers…” FG2-P7
Facilities	Access to facilities needed for prescribing. Includes drug charts, notes etc	“Still on paper. So sometimes it's just physically getting hold of the flaming drug chart.” FG1-P3
		“we have a very clear process in the trust, I get my pads from a… …lockable drawer from a named person, everything’s secure where I work, so I've got no problems.” FG2-P1
IT	Access to IT. Integration, or lack of, across areas. Issues using IT	“…the IT infrastructure in our place is just, it's just woeful…” FG1-P3
		“we also share in, in our trust, it’s community and acute services, and I have access to the electronic patient record that's used in the hospital, which I can also add records on, if I'm managing a patient that's also managed by the respiratory consultant, so we've got a really seamless um patient record…” FG2-P1
**Patient care**	Overarching theme exploring the use of NMP in patient care	
Impact on patients	Direct or indirect impact on patients and patient care	“…make sure it's happening in a time efficient manner, so patients are, you know, getting the prescriptions when they need because, particularly with the home care prescriptions as you have to work a month ahead.” FG1-P2
		“For me, it’s definitely reduced the time to treat, so before we’d have to write, request from GPs to prescribe inhalers or, urgent medications, whereas now writing a script in the patient's home, it’s just so much quicker.” FG2-P1
Quality & Safety	NMP improving quality and safety of prescribed medicines for the patient. Links to ‘Governance’ theme	“I guess for the inpatient side, um, when I do more of that, I guess more of patients are more likely to be started on the appropriate anticoagulation, at the right dose, etc.” FG2-P2
		“I suppose, and I probably said this, I think the probably the influence I have on the consultants is their, is their…, you know, assessing the use of their pain meds now, and is that appropriate. … … whereas consultants, if that's been their practice for years and years and years, it, you know, it wouldn’t, er wouldn't change unless it was challenged and I think we've got a good environment now that we can, we can have those discussions. … … And I think we bring a more balanced view possibly (nods from FG2-P4).” FG2-P5
Stopping	Function as NMP to stop medication/refer back to main prescriber/GP	“And also, that they get the medication they need stopping, stopped a lot quicker (nods from FG2-P4, FG2-P5 and FG2-P6).” FG2-P3
		“… we do more deprescribing now so I'm using it a lot less and when I do use it, it's more to give advice to GPs on how to maybe rationalize medication more than anything.” FG2-P5
**Orphan themes**	These themes do not fall easily under one overarching theme. They may cross link to several other themes or stand alone	
Conflict of interest	Theme highlighting conflicts of interest identified by participants	“…they don't want to push the case too hard, because they also want additional consultants, and they feel if they're saying I can do it than that weakens their case. So, it's almost like a conflict of interest there at the moment.” FG2-P7
		“But I suppose, even, even with herself, there is a conflict of interest because if I'm off prescribing that’s time taken away from delivering our service which she needs to manage.” FG2-P7
Covid	Impact that Covid-19 pandemic has had on NMP and /or their prescribing practice	“…particularly with Covid that aspect of the service has become more and more NMP led. I think at one point I was actually the only prescriber prescribing for that group of patients.” FG1-P2
		“my most recent experience was during Covid and being redeployed to wards for a couple of, well it was eight weeks, and you know generally junior medical staff did receive the prescribing well” FG1-P2
Good advice	What advice would the participants give new or prospective non-medical prescribers	“I would say you need to have decided with your organization where you're going to use it before you do the course, because otherwise you end up kind of stuck in limbo maybe without either the time or a role to use it.” FG2-P2
		“I think it’s really important that it's not just an additional duty that you take on as part of your role, have a dedicated area, have that time, time carved out so you can actually carry out that role.” FG2-P6
		“Don't be afraid to ask for help. You’re independent, but you're not alone.” FG1-P3
Undergrad Px	Prescribing taught at undergraduate level. Preparedness of new prescribers to take on role. Impact on rest of service	“…I share both your concerns, that I think at the moment the undergrads coming out too green to be independently prescribing and what we've already discussed about being not just the prescriber in, on paper, but actually it changes your role and becoming far more embedded into your team.” FG1-P3
		“…having undergrads coming out as prescribers, from a trust point of view, they're just going to be really expensive junior doctors, aren't they?” FG1-P3
		“…I think that when you’ve got a lot of junior people applying for jobs and you always say ‘Where do you see yourself in five years’, and they always say they want to do the prescribing course and you think, well, who’s going to be left just to do the day job, if everybody sees themself as a prescriber…” FG2-P4
		“…they're not really taught an awful lot about medications, never mind prescribing at undergraduate level so I think that’s got a long way to come.” FG2-P1

*ACP* advanced clinical practitioner, *CPD* continuing professional development, *GP* general practitioner, *IT* information technology, *MDT* multidisciplinary team, *NMP* non-medical prescriber, *TTO* ‘to take out’ – discharge medication

The five themes identified were ‘Staff’, ‘Self’, ‘Governance’, ‘Practical aspects’ and ‘Patient care’. Some subthemes did not fall easily under any of these themes, rather they linked disparate themes or subthemes, and are described as orphan themes. These were ‘Conflict of interest’, ‘Covid’, ‘Undergraduate prescribing’, and ‘Good advice’.

### Staff

This was the most frequently mentioned theme, particularly in relation to the clinical team but also to managers. The theme described the relationship between participants and senior and junior medical staff as well as other team members. Differences were highlighted in interactions between participants and senior or junior medical staff. The role within the clinical team was described and who lead on decision making. A lack of awareness of non-medical prescribing was identified by some, mainly physiotherapist, participants. Managers who prescribed were more supportive compared with non-prescribing managers, who may be unaware of prescribing governance issues. The ‘Managers’ subtheme linked to ‘Training’ and ‘CPD’ through the provision of funding and time.

### Self

This was the second most important theme, describing the participants’ practice. It encompassed the role prescribing took within their job and, for some, the challenges associated with incorporating this into their existing role, as well as prescribing within their personal competence, and support they gained from others, such as the clinical team. The theme highlighted training aspects including access to, and skills gained on, the course. The ‘Prescribing role’ subtheme linked to the ‘Stopping’ subtheme as part of ‘Patient care’.

### Governance

This theme incorporates aspects such as policies and guidelines supporting NMP, organisation NMP registers, formulary and continuing professional development (CPD). Participants highlighted other policies affecting their practice, including accountability for patient care, which may influence senior medical approach to non-medical prescribing. Two minor subthemes were identified, which were profession specific: ‘Legislation’ affecting physiotherapists and ‘Second check’ affecting pharmacists.

### Practical aspects

This theme incorporates those resources required to undertake prescribing, such as access to clinic rooms, information technology, appropriate budget and administrative support. Administration time was built into the roles for physiotherapist participants, whereas pharmacist participants described a lack of provision for administration time.

### Patient care

This theme incorporates aspects of patient care including the impact on patients by ensuring prescriptions were appropriate and completed in a timely manner. Other benefits included improvements in quality and safety for example by stopping inappropriate medication and having sufficient clinic time to check adherence. ‘Quality and safety’ linked with ‘Governance’.

### Orphan themes

Two of these themes were only mentioned by pharmacist participants and they were ‘Conflict of interest’ and ‘Undergraduate prescribing’. Several participants highlighted the impact the Covid-19 pandemic had on their practice and the final theme collated the advice the participants would give to new prescribers.

## Discussion

This study enabled an in-depth investigation of issues affecting pharmacist and physiotherapist non-medical prescribers. Participants’ lived experiences supported further exploration of the findings from a Delphi study [15]. Five themes, describing the experiences of the participants were identified.

### Themes

The theme of “Staff” reflected the previous Delphi findings whereby the clinical team (medical, nursing and peer support) accounted for approximately 40% of factors affecting NMP achieving consensus [15], and further confirming the role of medical professionals and colleagues in supporting NMP, identified in the preceding review [14]. This is unsurprising as all participants described working collaboratively to share the patient caseload, within a multidisciplinary team usually led by a medical professional. Traditionally senior medical staff were accountable for the patient’s care, and team members had closely defined roles. More latterly the move has been towards advanced practice in the non-medical professions, to develop a flexible workforce that is able to adopt innovative ways of working. This was described in the 2017 draft workforce strategy, which highlighted the increasing demand on the NHS, and the limited number of clinicians to provide care [36], and which built on earlier work such as developing primary care services [9, 37]. In addition, NMP courses require the trainee to complete a period of practice-based training supervised by an experienced prescriber. Until recently all regulatory bodies required this trainer to be a member of the medical profession, fostering closer links between trainer and trainee, which many participants commented on.

The “Self” theme, accounting for approximately a quarter of all references, focused on the “Prescribing role”, the role that prescribing had within the participant’s job and whether prescribing was integral to that role. All prescribers are required to prescribe within their scope of practice and the prescriber’s role implicitly defines that scope, together with guidance from regulatory and professional bodies [38–40]. Some pharmacist prescribers described challenges when prescribing had been added into their existing role, implying that for this group, the potential impact of prescribing had not been fully considered.

The“Practical aspects” and Governance” themes together highlighted the importance of ensuring adequate facilities for the prescriber, and a strong governance framework to support their prescribing practice. Covid-19 was found to affect some prescribers, either by altering how they practice, or by temporary changes to their role, as found by the “Covid-19″ theme. However, changes brought about by the Covid-19 pandemic also appeared in the “CPD” theme, with many participants describing online conferences and meetings becoming routine practice; enabling participation by a wider audience.

The relatively limited number of references to patient care may be considered surprising when compared with the Delphi study, where the top ranked statement concerned the effectiveness and benefits of prescribing for patients [15]. However, this finding partially reflects the different research methods, with Delphi seeking consensus whereas focus groups enable deeper exploration of lived experiences of the participants. It also reflects the topics chosen for discussion, which were those where there were areas of potential disagreement between the two prescribing professions, and hence patient care was a subsidiary aspect of the discussion.

### Inter-dependencies

The previous review exploring barriers and facilitators to non-medical prescribing identified that many of the factors involved were inter-dependent [14]. The experiences of the participants in this study supported this finding, with the important secondary co-dependencies depicted in Fig. 2. The “Quality and safety” theme was interdependent with all aspects of the “Governance” theme, resulting in improved care for patients. For example, participant FG2-P5 described constructive discussions with senior medical staff, informed by policies and guidance, resulting in team-wide changes in prescribing practice and improved patient care. For pharmacy managers, there was an implicit conflict between service delivery and governance, inferred by the “Second check” theme. Pharmacists are experts in medicines [41]; clinically screening prescriptions, the so called ‘second check’, to ensure appropriateness for the patient. Pharmacy managers are required to maintain the governance structure surrounding medicines supply, within a limited staffing establishment, and this can result not only in limiting time for pharmacist prescribing, but also difficulty in providing the second check. Evidence indicates that pharmacist prescribers make fewer errors than medical staff [42], but pharmacist participants perceived that they had been left without an important safety net. Further co-dependencies described by participants included the impact on senior medical staff of policies regarding patient accountability, with concern by some senior medical staff that they were accountable for the non-medical prescriber’s actions. This lack of clarity regarding accountability was identified in the previous review [14]. The prescribing competency framework for all prescribers states that the prescriber is accountable for their prescribing decisions [39], however if a policy regarding patient accountability states that the consultant is responsible for the actions of their entire team, then this could result in confusion.

### Inter-professional differences

Differences were highlighted between professions, many of which could be anticipated from the way in which each profession traditionally works. For physiotherapists, prescribing forms another treatment option when caring for patients, fitting in to existing roles such as in musculoskeletal clinics [43], whilst also supporting the development of new roles based on existing skills [10]. For the secondary care pharmacist participants, prescribing in many instances was in addition to their existing role, without due consideration to restructuring job plans to allow sufficient time. Consequently, physiotherapist participants felt well-supported for administration time, whereas for the pharmacist participants, unless expressly included in their job plan, administration time was a source of stress. Similarly, pharmacist participants, used to working in a team, described a team approach to decision making, compared with physiotherapists, used to planning treatment courses for patients, who were more inclined to make their own decisions.

For the physiotherapist participants, the choice of medicines that they can prescribe is limited by their professional scope of practice and legislation [38, 44], compared with pharmacists who can prescribe any medication, except certain drugs for the treatment of addiction [45, 46]. For the physiotherapists, probable changes in controlled drug legislation have the potential to influence how advanced practice roles develop, particularly if physiotherapists continue to have restricted access to controlled drugs [47]. One physiotherapist participant described the constraints imposed by controlled drug legislation in chronic pain management, but commented that current guidance was moving away from drug treatment and hence expanding the choice of controlled drugs physiotherapists could prescribe may have limited impact in their case [47, 48].

Physiotherapist participants were more likely to describe lack of awareness of physiotherapist prescribing by the clinical team, than pharmacist participants. This reflects both the relatively short time span in which physiotherapists have had prescribing rights (independent prescribing rights since 2013) and the small numbers registered as prescribers (1017 independent prescribers in 2019) [17, 19]. In comparison, pharmacists gained independent prescribing rights in 2006, with 8077 independent prescribers on the register in 2019 [49, 50].

Planned changes in pharmacist pre-registration training, including at undergraduate level, will result in newly registered pharmacists registering as independent prescribers [51]. Pharmacist participants expressed concerns about this development, including detraction from training aspects and potential exacerbation of prescribing errors, as previously identified with junior medical staff [52]. The participants placed their views in the context of their own prescribing training, highlighting the struggle that less experienced pharmacists had with the course, and commenting that routine pharmacy work still needed addressing. However, the development is in line with the Carter report and draft workforce strategy, which both envisaged a clinical pharmacy workforce, with pharmacy technicians adopting some of the traditional pharmacist roles [36, 53]. The concerns expressed by pharmacist participants regarding time pressures to complete their tasks suggest that advanced pharmacy technician roles, which would release pharmacist time for prescribing, have still to be adopted.

Trustworthiness of the data is supported by the approach to analysis. Full, in-depth discussion of the findings by all authors, with challenge of the derived themes to ensure that they reflected the participants experiences was undertaken. The differences in background and experiences of the research team composition ensured that EGC’s longstanding prescribing experience in critical care, and possible preconceptions, were counterbalanced by the other team members, who were non-prescribers but clinicians in both physiotherapy and pharmaceutical fields. Data saturation was achieved, with the themes and main subthemes identified by each focus group and profession. This is supported by the answers to the final question regarding advice to new prescribers, added as a positive end note to each session. No new ideas were articulated but participants emphasised the need for a prescribing role, ensuring facilities were in place beforehand, asking for advice and not being pressurised to prescribe medication that they deemed outside their personal competence.

### Strengths and limitations

The study allowed in-depth discussion of issues affecting pharmacist and physiotherapist prescribers, with ideas developed by the participants throughout the discussion. Participants drew on their experiences to describe issues affecting them, allowing a greater understanding of the background and contributory factors. As the themes were derived directly from these lived experiences, they acquired content and face validity.

The virtual platform, with choice of dates and times, allowed participants to join who may otherwise have been unable to because of constraints such as work commitments.

The Covid-19 pandemic limited recruitment: in particular fewer physiotherapist participants were recruited than planned. However, findings appeared unaffected with no new themes emerging from the second focus group. This supports the assertion that data saturation was achieved for the major themes identified.

It is acknowledged that recruitment may have been enhanced by widening the geographical catchment area. However, it was possible that some of the variation seen in the previous Delphi results [15] may have arisen from the wide range of practice and geographic areas in which participants were employed. Therefore a deliberate decision was made to limit recruitment to pharmacist and physiotherapist prescribers working in the NHS West Midlands area (either primary or secondary care), to reduce the risk of introducing variability into the findings.

## Conclusion

The key finding from this study related to the theme of collaborative working with the clinical team; emphasising the impact this has on successful implementation of NMP. When their role was specifically designed to include prescribing, this was a benefit for pharmacist participants. Multiple factors contribute to the themes of governance, practical aspects and patients, and each factor is important for successful implementation of NMP. Crucially, the identified themes and subthemes cannot be considered in isolation but are inter-dependent on each other.

Differences between the professions were illustrated from the analysis, most reflecting the way each profession practises and, for pharmacists, the way that prescribing has been introduced into their role. For the pharmacists, managers need to address the skill mix to enable pharmacist prescribers to practise with support.

To ensure NMP is fully enabled, all aspects must be fully scoped before recruiting or training a non-medical prescriber. Failure to do so may limit full utilisation of prescribing skills and result in a poorly motivated workforce.

## Supplementary Information

**Additional file 1.** Focus group topic guide.

**Additional file 2.** Consolidated criteria for reporting qualitative studies(COREQ): 32-item checklist.

## Data Availability

The datasets used and/or analysed during the current study are available from the corresponding author on reasonable request.

## Abbreviations

CPD: Continuing professional development
IPA: Interpretative Phenomenology Analysis
NHS: National Health Service
NMP: Non-medical prescribing
UK: United Kingdom
