# Supplementary material for: Exploring the barriers and facilitators to non-medical prescribing experienced by pharmacists and physiotherapists, using focus groups
Source: BMC Health Serv Res. 2022 Feb 18;22:223. doi: 10.1186/s12913-022-07559-5 (PMC8854478; doi:10.1186/s12913-022-07559-5)
Supplement: Supplementary file 1 — Additional file 1. Focus group topic guide. [file 12913_2022_7559_MOESM1_ESM.docx]

# Additional file 1

**Focus group topic guide**

Preamble/introduction

- Thank participants for logging in
- Introduce moderator role, to conduct the meeting
- The session will be recorded so that the full discussion is captured, do all participants consent to that?
  - As it is audio recorded, please only one person speak at a time
- Background to research.
- Investigation into the utilisation of non-medical prescribing by pharmacists and physiotherapists.
- Follow on from Delphi study which investigated barriers and facilitators experienced by physiotherapist and pharmacist participants to the utilisation of NMP.
- 29 statements describing facilitators and barriers to prescribing reached consensus, but there were differences between the professional groups regarding the relative importance of each of these.
- Focus group aim is to explore these findings in more depth from each profession’s perspective (i.e. pharmacist and physiotherapist).
- Findings will form part of doctoral research thesis by moderator.
- Intention is to publish results
- Emphasise that it is participants views that are wanted, all opinions are valued and there are no right or wrong answers
- Participants do not have to discuss information that they are uncomfortable with.
- If a serious patient safety issue is disclosed, then confidentiality will be broken so that the information can be reported to the relevant authority (as stated in the PIS).
- Participation is entirely voluntary
- Discussion will remain confidential, and the transcript of the meeting will be anonymised before analysis. Only anonymous quotes will be used in any thesis or publication.

**Start recording!**

**Introductory question**

Can everyone introduce themselves, and just say a little bit about **where** they use their prescribing?

**Opening topic**

Can you say something about/describe how you use NMP?

What impact has it had for you in caring for your patients?

**Influence of medics**

We’d like to explore your relationships with other staff now

Can you describe your working relationship with medical staff?

Probe – please give an example

Issues may include: senior and junior medical staff, mentor, included in team, isolated

Probe negative and positive comments to see if others have a similar viewpoint

What effect has this relationship had on your practice as an NMP?

Probe – please give an example

Issues may include: mentor, support, encouragement, negativity, blocking, enabling, giving confidence

Probe negative and positive comments to see if others have a similar viewpoint

Is there anything else you wish to add about your working relationships with medical staff?

Moderator to summarise discussion regarding medics

**Influence of managers**

How about your managers, how would you describe their attitude to NMP?

Probe – please give an example

Issues may include: support for course, support for CPD, support for NMP in general, job plan,

Probe negative and positive comments to see if others have a similar viewpoint

What effect has this relationship had on your practice as an NMP?

Probe – please give an example

Issues may include: support, encouragement, negativity, blocking, enabling

Probe negative and positive comments to see if others have a similar viewpoint

Is there anything else you wish to add about your working relationships with managers?

Moderator to summarise discussion regarding managers

**Organisational aspects**

Turning to the organisations you work in now

What practical aspects have influenced your ability to prescribe, for example access to facilities?

Probe – please give an example

Issues may include: joining the NMP register, policies NMP and other, scope of practice, limitations, access to prescriptions/electronic prescribing, access to patient records, access to clinic rooms, time, access to information to support prescribing

Probe negative and positive comments to see if others have a similar viewpoint

What about the way you work? Some of you describe working in a team, some on your own, what impact has the way you work had on your prescribing practice?

Probe – please give an example

Issues may include: isolation, freedom to make decisions, constrained by team, supported by team, advantages, disadvantages

Probe negative and positive comments to see if others have a similar viewpoint

Is there anything else you wish to add about the practical side of prescribing?

Moderator to summarise discussion regarding medics

**Final question**

What one bit of advice would you give to new non-medical prescribers to enable them to utilise their new qualification?

**Closing stage**

Summarise discussion

Check that nothing has been missed that participants feel should have been included

**Stop recording**

Thank participants for their help
